# Fra-1 Inhibits Cell Growth and the Warburg Effect in Cervical Cancer Cells via STAT1 Regulation of the p53 Signaling Pathway

**DOI:** 10.3389/fcell.2020.579629

**Published:** 2020-09-30

**Authors:** Manying Zhang, Lin Liang, Junyu He, Zhengxi He, Chunxue Yue, Xi Jin, Mengxiang Gao, Songshu Xiao, Yanhong Zhou

**Affiliations:** ^1^Hunan Cancer Hospital and The Affiliated Cancer Hospital of Xiangya School of Medicine, Central South University, Changsha, China; ^2^NHC Key Laboratory of Carcinogenesis, Hunan Cancer Hospital and The Affiliated Cancer Hospital of Xiangya School of Medicine, Central South University, Changsha, China; ^3^Department of Gynecology and Obstetrics, The Third Xiangya Hospital, Central South University, Changsha, China

**Keywords:** Fra-1, STAT1, cervical cancer, cell growth, Warburg effect

## Abstract

The oncogenesis of cervical cancer is a multi-factor and multi-step process, and major risk factors include oncogene activation with tumor suppressor gene inactivation, viral factors, and immune factors. For example, the human papillomavirus (HPV) has been linked to the occurrence of cervical cancer. At present, the pathogenesis of cervical cancer remains unclear. Fra-1 (Fos-related antigen 1, also known as FOSL1) is a member of the Fos family and an important nuclear transcription factor that regulates normal cell growth, differentiation, and apoptosis. In the present study, we found that Fra-1 inhibited the proliferation of cervical cancer cells while also promoting apoptosis and affecting cell cycle distribution. Moreover, Fra-1 up-regulated STAT1 expression and modulated p53 signal pathway activity in cervical cancer cells. Overexpression of Fra-1 inhibited cell senescence by altering sirtuin 1 (SIRT1) expression in HeLa cells, and Fra-1 overexpression restored mitochondrial disorder and suppressed metabolic reprogramming in HeLa cells. Silencing of STAT1 impaired the inhibitory effect of Fra-1 on cervical cancer cell growth, while knock-down of STAT1 reversed the effect on cell senescence and mitochondrial dysfunction caused by Fra-1 in HeLa cells. Silencing of STAT1 also recovered metabolic reprogramming in cervical cancer cells. In summary, our results show that Fra-1 inhibited cervical cancer cell growth and the Warburg effect via STAT1-mediated regulation of the p53 signaling pathway.

## Introduction

Among Chinese women aged 15–44 years, cervical cancer is the second most common cancer and the most common gynecological malignancy (Liou et al., 2016; Gu et al., 2017; Vu et al., 2018). The typical age range for onset of carcinoma *in situ* is 30–35 years, and that for invasive cancer is 45–55 years. In recent years, the incidence of cervical cancer in younger patients has increased (Schwarz et al., 2009; Cao et al., 2010; Liu et al., 2015). Most cervical cancer cases (∼99.7%) are accompanied by high-risk human papillomavirus (HPV) infection, and persistent infection with high-risk HPV has been shown to be a major risk factor for cervical cancer. The incubation period for HPV is long, with oncogenesis commonly occurring 8–10 years after infection. While HPV infection is a known cause of cervical cancer, it does not fully explain the occurrence of cervical cancer. Other factors are important in the malignant transformation of high-grade HPV infection (Kaliff et al., 2018; Farazi et al., 2019; So et al., 2019). The key events leading to oncogenesis are mediated by many factors, which need to be further understood (Ramdass et al., 2013; Karim et al., 2018; Mirbahari and Sadeghi, 2018). Many gaps in knowledge regarding cervical cancer pathogenesis and the corresponding treatment mechanism remain to be filled.

Tumor cells commonly achieve enhanced proliferation, growth, survival, and long-term maintenance via alteration of their metabolism. The rate of glucose uptake and lactate production is increased dramatically in many tumors cells, which requires sufficient oxygen and fully functioning mitochondria. This is known as the Warburg Effect and has been explored extensively (Nagao et al., 2019), since Otto Warburg first described this phenomenon in the 1920s, with studies producing both supportive and opposing evidence (He et al., 2016).

Fra-1 (Fos-related antigen 1, also known as FOSL1) is a member of the Fos family and an important nuclear transcription factor that regulates normal cell growth, differentiation, and apoptosis (Annis et al., 2018; Xu et al., 2018). Fra-1 is highly expressed in many malignant tumors and plays an important role in cell transformation, proliferation, invasion, and metastasis (Annis et al., 2018; Wang et al., 2018). Fra-1 activity is regulated at both the transcriptional and translation levels (Xiao et al., 2015; Belguise et al., 2017). Our preliminary studies suggested that Fra-1 can inhibit the proliferation of cervical cancer cells (Xiao et al., 2015), but the underlying mechanism remained unclear. Therefore, in the present study, we investigate the effects and possible mechanisms of Fra-1 on the proliferation, apoptosis, and senescence of cervical cancer cells.

Signal transducer and activator of transcription 1 (STAT1) has been reported to act as a tumor suppressor. Studies have shown that STAT1 plays an important role in apoptotic and anti-apoptotic signaling, demonstrating that it can regulate apoptosis by inhibiting non-transcriptional mechanisms such as anti-apoptotic protein nuclear factor (NF)-kappa B (Zhang et al., 2018). In renal cell carcinoma cells, down-regulation of STAT1 expression can slow cell growth (Ah-Koon et al., 2016). Additional research has linked STAT1 with the development of many malignant tumors, including breast cancer, myeloma, and renal cancer (Suyama et al., 2016; Chen et al., 2017; Qu et al., 2017; Josahkian et al., 2018). Specifically, STAT1 was shown to regulate p53 activity by inducing phosphorylation of p53 (Chen et al., 2017). Through such interaction with p53, STAT1 promotes apoptosis, and STAT1 also interacts with the p53 inhibitor MDM2 (Chen et al., 2017). Therefore, we explored whether the potential effects of Fra-1 on cervical cancer cell growth and the Warburg effect in these cells are mediated by STAT1 regulation of the p53 signaling pathway.

Here we first investigated the effect of Fra-1 on cell growth and the Warburg effect in cervical cancer cells. Then, we determined the influence of Fra-1 on STAT1 expression. Finally, we confirmed that the inhibition of cell growth and the Warburg effect in cervical cancer cells by Fra-1 is mediated via STAT1.

## Materials and Methods

### Cell Culture

HeLa cells, a human cervical cancer cell line, were purchased from the ATCC (Manassas, VA, United States) and maintained by our laboratory. The HeLa cells were cultured in RPMI-1640 supplemented with 10% fetal bovine serum (FBS; Gibco Life Technologies, Grand Island, NY, United States) in a 5% CO_2_ incubator at 37°C.

### Construction of pEGFP-N1-Fra-1 Vector and Cell Transfection

The coding region of the Fra-1 gene was amplified by polymerase chain reaction (PCR). The primer sequences for the Fra-1 gene were 5′-atactcgaatgaacctggccatcagcat-3′ and 5′-gcggaattctcacagggacatgaaatccg-3′. The conditions for PCR amplification were one cycle for 5 min at 94°C; 30 cycles for 45 s at 94°C, 45 s at 55°C, and 90 s at 72°C, and a final cycle for 10 min at 72°C. The coding region fragments of the Fra-1 gene were cloned into the TA vector (Promega, Fitchburg, WI, United States) for transformation in *Escherichia coli* JM109 (Takara, Dalian, China). In the TA vector, restriction endonuclease xhoi and ecor1 (Promega) were used to cut the required DNA fragments, which were then subcloned into the pEGFP-N1 vector. The accuracy of the cloned sequence was confirmed by DNA sequencing using an ABI 3730 instrument (Applied Biosystems, Foster City, CA, United States).

To construct a stable cervical cancer cell line overexpressing Fra-1, we transfected HeLa cells with the pEGFP-N1/Fra-1 or pEGFP-N1 (blank control vector) using Lipofectin (Invitrogen, Carlsbad, CA, United States) according to the manufacturer’s instructions as described previously (Xiao et al., 2015). This was followed by G418 selection. Western blotting was performed to detect the expression level of Fra-1 protein to verify successful establishment of the stable cell line.

### Colony Formation and CCK8 Assay

Cell proliferation was evaluated using colony formation and CCK8 assays. For the colony formation assay, 1,000 cells were seeded in each well of six-well plates and cultured at 37°C. After 14 days in culture, the cells were fixed in 4% paraformaldehyde solution and stained with 0.1% crystal violet solution. Colonies were counted and photographed. For the CCK8 assay, cells were seeded in 96-well culture plates at a density of 1,000 cells/well in 200 μl medium. After culture at 37°C for 6 days, we analyzed the cell density in each well every 24 h using the 7Sea-Cell Counting Kit (7Sea Biotech, Shanghai, China). Briefly, 20 μl of CCK8 solution was added to each well and incubated for 2 h at 37°C before the absorbance at 450 nm was measured by a Paradigm Detection System (Beckman Coulter, Brea, CA, United States).

### Flow Cytometric Analysis of Cell Cycle Distribution and Apoptosis

Prior to flow cytometric analyses, cells (5 × 10^5^/well) were seeded in 6-well cell culture plates and cultured in a humidified atmosphere of 5% CO_2_ at 37°C for 24 h before harvesting with 0.5% trypsin and centrifugation. For cell cycle analysis, cells were fixed in ice-cold 70% ethanol overnight at 4°C. The fixed cells were then resuspended in 500 μl phosphate-buffered saline (PBS) to which 10 μl RNase A was added for a 5-min incubation followed by three washes with cold PBS. Next, 10 μl propidium iodide (PI) solution was added to the resuspended cell solution for incubation at 4°C for 30 min. Finally, the cells were washed twice with PBS before flow cytometric analysis of cell cycle distribution. For flow cytometric detection of apoptotic cells, the Hoechst 33342/PI Apoptosis Assay Kit (BestBio, Shanghai, China) was used. Briefly, the harvested cells were washed twice with ice-cold PBS and resuspended in 500 μl staining buffer. Then, the resuspended cell solution was incubated with 5 μl Hoechst 33342 and 5 μl PI solution in darkness for 20 min at 4°C. Finally, the cells were washed twice with PBS before flow cytometric analysis of cell apoptosis. All flow cytometric analyses were performed using a MoFlo^TM^ XDP High-Performance Cell Sorter (Beckman Coulter). The data for cell cycle distribution and apoptosis were analyzed using Summit v5.2 Software (Beckman Coulter).

### Quantitative Real-Time PCR

The total RNA of HeLa cells was isolated using TRIzol reagent (Invitrogen), and cDNA was then generated by reverse transcription from 1 μg total RNA using the HiScript II Q RT SuperMix for qPCR (Vazyme, Nanjing, China). Quantitative real-time PCR was carried using ChamQTM SYBR^®^ qPCR Master Mix (Vazyme, Nanjing, China). The primers used for real-time PCR are listed in Table 1.

**TABLE 1:** List of the human-specific primer sequences used for qPCR analyses.

**Gene**	**Primer Sequence**		**T_*m*_ (°C)**
	**Forward**	**Reverse**	
STAT1	5′-ttcaggaagacccaatccag-3′	5′-tgaatattccccgactgagc-3′	60
p53	5′-gttccgagagctgaatgagg -3′	5′-tctgagtcaggcccttctgt-3′	60
Bcl-2	5′-atgtgtgtggagagcgtcaa-3′	5′-acagttccacaaaggcatcc-3′	60
p38	5′-tgactcagatgccgaagatg-3′	5′-atcataaggatcggccactg-3′	60
p21	5′-ggaagaccatgtggacctgt-3′	5′-ggattagggcttcctcttgg-3′	60
MDM2	5′- ggtgggagtgatcaaaagga-3′	5′-gtggcgttttctttgtcgtt-3′	60
CDK4	5′-cccgaagttcttctgcagtc-3′	5′-ctggtcggcttcagagtttc-3′	60
Cyclin D1	5′- cctgtcctactaccgcctca -3′	5′- cacctcctcctcctcctctt -3′	60
IL-6	5′-actcacctcttcagaacgaattg-3′	5′-ccatctttggaaggttcaggttg-3′	60
IL-8	5′-actgagagtgattgagagtggac-3′	5′-aaccctctgcacccagttttc-3′	60
GLUT1	5′-gcgggttgtgccatactcatgacc-3′	5′-aggccacaaagccaaagatggcc-3′	59
HK II	5′-gagccaccactcaccctact-3′	5′-ccaggcattcggcaatgtg-3′	60
LDHA	5′-ttgacctacgtggcttggaag-3′	5′-ggtaacggaatcgggctgaat-3′	60
β-tubulin	5′-aagatccgagaagaataccctga-3′	5′-ctaccaactgatggacggaga-3′	60

*STAT1, signal transducer and activator of transcription 1; Bcl-2, B-cell lymphoma 2; CDK4, cyclin dependent kinase 4; IL-6, interleukin 6; IL-8, interleukin 8; Glut1, glucose transporter 1; LDHA, lactate dehydrogenase A.*

### Detection of Cellular Senescence by β-Galactosidase Staining

HeLa cells were washed three times in six-well plates with 1 × PBS and fixed with 4% paraformaldehyde at room temperature for 15 min. The plate was rinsed two times with 1 × PBS, and 1 ml of staining solution was added to each well. The plate was incubated overnight at 37°C without CO_2_. Cellular senescence was detected using a Senescence-associated β-Galactosidase Staining Kit (C0602, Beyotime, China). On images captured with a light microscope (CKX41, Olympus, Japan), β-galactosidase–positive cells (blue staining) were considered senescent cells.

### Intracellular Ca^2+^ Concentration Assay

The fluorescent dye Fura-2 AM (Beyotime Institute of Biotechnology, Haimen, China) penetrates the cell membrane and binds calcium ions to produce strong fluorescence under 330–350 nm excitation light and weak fluorescence under 380 nm excitation light. The ratio of 340 and 380 nm fluorescence is routinely used to evaluate the intracellular calcium concentration. Fura-2 staining was performed according to the manufacturer’s instructions in the present study. Afterward, the cells were washed twice and resuspended in PBS for fluorescence-activated cell sorting (FACS) analysis (Beckman Coulter). The Ca^2+^ concentration data were analyzed using Summit v5.2 software (Beckman Coulter).

### Intracellular Reactive Oxygen Species (ROS) Measurement

HeLa cells were trypsinized, counted, and then resuspended in culture medium (1 × 10^6^/ml/tube). To 1 ml of cell suspension, we added 1 μl of 10 mM DCFH-DA active oxygen fluorescence probe solution (Applygen, Beijing, China). For the negative control group, 1 μl dimethyl sulfoxide (DMSO) solution was added, and for the positive control group, 1 μl active oxygen positive control solution and 1 μl of 10 mM DCFH-DA active oxygen fluorescence probe solution was added. For all groups, the mixtures were incubated at 37°C for 20 min, with pipetting of the probe solution and mixing every 3–5 min to ensure good distribution of the cells. After washing with 1 × PBS solution two or three times, the cells were resuspended in a proper volume of 1 × PBS (generally 400–500 μl) for testing of reactive oxygen species (ROS) levels. The cells were then washed twice with cold PBS and resuspended in PBS for analysis of the intracellular ROS concentration by FACS (Beckman Coulter).

### Determination of Mitochondrial Membrane Potential Using JC-1

The effect of Fra-1 of mitochondrial membrane potential was assessed by flow cytometry using the sensitive and relatively mitochondrion-specific lipophilic cationic probe fluorochrome JC-1. JC-1 accumulates to form J-aggregates and emits red fluorescence in mitochondria with a high membrane potential, yet dissociates into monomers and emits green fluorescence in mitochondria that have lost their cross-membrane electrochemical gradient. The groups of HeLa cells were suspended in 1 ml warm staining buffer at approximately 1 × 10^6^ cells/ml and incubated at 37°C for 5 min. Then 1 μl of 2 mM JC-1 (2 μM final concentration; MultiSciences Biotech, Co., Ltd., Hangzhou, China), and the cells were incubated at 37°C in 5% CO_2_, for 15–30 min. Afterward, the cells were pelleted by centrifugation and resuspended by gentle tapping of the tubes, and then 500 μl PBS was added to each tube. Mitochondrial membrane potential was assessed by FACS (Beckman Coulter).

### Small Interfering RNA-Based Silencing of STAT1

The siRNAs were designed and synthesized by Guangzhou RiboBio (RiboBio, Guangzhou, China). The siRNAs targeting on STAT1 gene were designed and synthesized, and the most effective siRNA (STAT1) identified by qPCR was applied in further experiments. Twenty-four hours prior to transfection, cells were plated onto a six-well plate (Greiner, Germany) at 40–60% confluence. Transfection was performed with Lipofectamine 2,000 (Invitrogen) according to the manufacturer’s protocol. The medium was replaced 4–6 h after transfection with new culture medium.

### Measurement of NAD^+^/NADH Ratio

To assess mitochondrial function by evaluation of the NAD^+^/NADH ratio, NAD^+^ and NADH were extracted from cells. For NAD^+^ extraction, 1 × 10^6^ cells were collected in a centrifuge tube. The supernatant was discarded, and 0.9 ml was added acid extract, followed by sonication of the solution for 1 min (intensity 40%, 4 s, stop for 2 s). The solution was then boiled for 5 min, cooled in an ice bath, and centrifuged for 10 min at 10,000 × *g* at 4°C. The supernatant was transferred to another new centrifuge tube to which an equal volume of alkaline extract was added to neutralize the solution. Centrifugation at 10,000 × *g* at 4°C for 10 min was repeated, and the supernatant was collected and stored on ice for testing. For NADH extraction, 4–5 million cells were collected in a centrifuge tube. The supernatant was discarded, 0.9 ml alkaline extract was added, followed by sonication of the solution for 1 min (40%, 4 s, stop for 2 s). The solution was then boiled for 5 min, cooled in an ice bath, and centrifuged for 10 min at 10,000 × *g* at 4°C. The supernatant was transferred to another new centrifuge tube to which an equal volume of acidic extract was added to neutralize the solution. Centrifugation at 10,000 × *g* at 4°C for 10 min was repeated, and the supernatant was collected and stored on ice for testing.

For testing of NAD^+^ and NADH levels, the solutions were centrifuged at 20,000 × *g* at 4°C for 5 min. The supernatant was discarded, and precipitation solution (Nanjing Jiancheng Bioengineering Institute, Nanjing, China) was added for measurement of the solution absorbance at 570 nm.

### Measurement of Lactic Acid Content and Production

Lactic acid content within the cells and within the media was detected in samples of approximately 5 × 10^5^ HeLa cells cultured for 48 h. The cells were homogenized by ultrasonic fragmentation, and the lactic acid measurement kit reagents (Nanjing Jiancheng Bioengineering Institute, China) were added according to the manufacturer’s instructions for sample loading. The absorbance in each well was measured at 530 nm using the Paradigm Detection Platform (Beckman Coulter). Lactic acid measurements were normalized to total protein concentration, which was tested using the BCA assay (Thermo Fisher Scientific), for each sample.

### Measurement of Glucose Concentration

HeLa cells were plated in 6-well culture plates (1 × 10^6^ cells per well) and incubated at 37°C in 5% CO_2_ for 24–48 h. When the cells reached approximately 80% confluency, the culture medium was transferred to a new centrifuge tube and centrifuged at 1,000 × *g* for 5 min at 4°C to remove insoluble materials. The supernatant was transferred to specific tubes for the detection of glucose concentration in the medium by the ADVIA 1,650 automatic biochemical analyzer (SIEMENS, Berlin, Germany). Meanwhile, the cells on the culture plate were trypsinized, washed with cold 1 × PBS, and resuspended in microcentrifuge tubes. After 3 min of ultrasonication on ice, the samples were centrifuged at 12,000 × *g* for 20 min at 4°C. This supernatant was then transferred to specific tubes for intracellular glucose concentration detection by the ADVIA 1,650 automatic biochemical analyzer. All values were normalized according to protein concentration, which was determined by the BCA assay.

### Measurement of Glutamine Production

For measurement of glutamine production, aliquots of 1 × 10^6^ HeLa cells were seeded and cultured for 24 h before harvesting, washing with cold PBS, and resuspension in 100 μl of Hydrolysis Buffer (BioVision, Milpitas, CA, United States). The cells were quickly homogenized by rapid pipetting up and down several times. The glutamine content was then assessed using the glutamine colorimetric assay kit according to the manufacturer’s instructions (BioVision, Milpitas, CA, United States) and normalized according to the protein concentration, which was determined by the BCA assay for each sample.

### Western Blotting Analysis

For western blot analysis of protein expression levels, HeLa cells were lysed in radioimmunoprecipitation (RIPA) buffer (CWBio, Beijing, China). Then 50 μg lysate was electrophoresed on 10% separation gel and transferred to a polyvinylidene difluoride membrane (HyClone Laboratories, Logan, UT, United States), which was sealed with 5% non-fat milk diluted in PBS-Tween20 for ∼2 h. Primary antibody solution was then added for incubation at 4°C for 12 h. The primary antibodies used were: anti-sirtuin 3 (SIRT3, dilution ratio: 1:500), anti-Mn superoxide dismutase 2 (MnSOD2, dilution ratio: 1:1,000), anti-isocitrate dehydrogenase 2 (IDH2, dilution ratio: 1:500), anti-liver kinase B1 (LKB1, dilution ratio: 1:500), anti-p-AMP-activated protein kinase (AMPK, dilution ratio: 1:1,000), anti-pyruvate kinase muscle isozyme 2 (PKM2, dilution ratio: 1:1,000), anti-phosphofructose kinase 1 (PFK1, dilution ratio: 1:1,000), anti-pyruvate dehydrogenase (PDH, dilution ratio: 1:1,000), anti-FOS like 1, AP-1 transcription factor subunit (FOSL1, Fra-1, Abcam, Cambridge, MA, United States, dilution ratio: 1:500), anti-signal transducer and activator of transcription 1 (STAT1, dilution ratio: 1:500), anti-c-Rel (c-Rel, dilution ratio: 1:1,000), anti-lactate dehydrogenase A (LDHA, dilution ratio: 1:1,000), anti-sirtuin 1 (SIRT1, dilution ratio: 1:500), anti- B-cell lymphoma-2 (Bcl-2, dilution ratio: 1:1,000), anti-NF kappa-B (NF-κB/p65, dilution ratio: 1:1,000, Immunoway Technology, Newark, DE, United States), anti-TP53 (p53, dilution ratio: 1:1,000), anti-cyclin-dependent kinase 4 (CDK4, dilution ratio: 1:1,000), anti-cyclin D1 (dilution ratio: 1:1,000), anti-p21 (dilution ratio: 1:1,000), anti-MAPK14/p38 (dilution ratio: 1:1,000), and anti-MDM2 (dilution ratio: 1:1,000) anti-p16 (dilution ratio: 1:1,000, Boster Technology, Wuhan, China). After three washes on a shaker with PBS-Tween20, the membranes were incubated with anti-rabbit or anti-mouse horseradish peroxidase (HRP)-conjugated secondary antibody (Santa Cruz Biotechnology, Santa Cruz, CA, United States, 1:3,000 dilution) for 1 h at 37°C). Finally, the protein bands were developed using the Luminata Forte western HRP substrate (Millipore, Billerica, MA, United States). Anti-β-tubulin (Santa Cruz Biotechnology) expression was used for normalization.

### Cervical Cancer Tumor Growth *in vivo*

The study protocol was approved by and conducted in accordance with the Committee of the Use of Live Animals in Teaching and Research at the Central South University. Twelve four-week-old BALB/c-nu female nude mice were purchased from the animal institutes of the Chinese Academy of Medical Sciences and Peking Union Medical College (CAMS and PUMC). We divided the mice into three groups (*n* = 4 each). HeLa cells transfected with the control vector (HeLa vector), HeLa cells transfected to overexpress Fra-1 (HeLa/Fra-1), and HeLa cells with overexpression of Fra-1 and silencing of STAT1 (HeLa/Fra-1/siSTAT1) of good cell status were inoculated into the right armpit of the mice in the three respective groups at a concentration of 6 × 10^6^ cells/150 μl. The mice were monitored twice weekly for palpable tumor formation and euthanized 15 days after transplantation for assessment of tumor formation. Tumors were measured using a Vernier caliper, weighed, and photographed.

### Statistical Analysis

All data were presented as mean ± standard deviation (SD) values obtained from three independent experiments and analyzed by one-way analysis of variance (ANOVA). Then two-way ANOVA followed by Tukey’s multiple comparison test for multiple comparisons was performed. GraphPad Prism 5.0 (GraphPad Software, La Jolla, CA, United States) was used for all analyses, and a value of *P* < 0.05 indicated a statistically significant difference.

## Results

### Fra-1 Inhibited Proliferation, Promoted Apoptosis, and Altered the Cell Cycle Distribution of Cervical Cancer Cells

**FIGURE 1:**
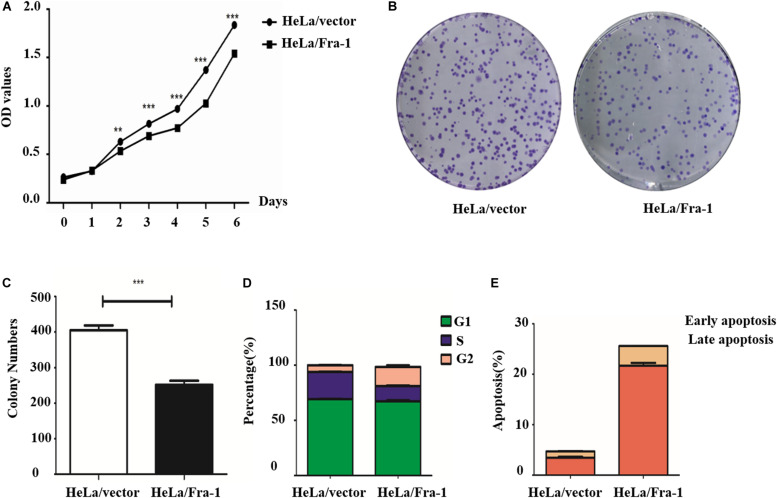
Fra-1 inhibited the proliferation and promoted apoptosis of cervical cancer cells. **(A)** Cell viability of HeLa cells transfected with control vector (HeLa/vector) and HeLa cells transfected with Fra-1 overexpression vector (HeLa/Fra-1) cells determined by CCK8 assay. **(B)** Colony forming ability of HeLa/vector and HeLa/Fra-1 cells. **(C)** Colony numbers formed by HeLa/vector and HeLa/Fra-1 cells. **(D)** Cell cycle distribution among HeLa/vector and HeLa/Fra-1 cells as determined by flow cytometry. **(E)** Flow cytometric analysis of apoptosis among HeLa/vector and HeLa/Fra-1 cells. Data means ± standard deviation (SD). Each representative experiment was repeated three times with similar results. ^∗∗^*P* < 0.01; ^∗∗∗^*P* < 0.001, *n* = 3.

Our previous research demonstrated lower Fra-1 expression in cervical carcinoma tissues compared to adjacent normal tissues. To further investigate this feature of cervical cancer cells, we established a stable Fra-1–overexpressing HeLa cell line using lentivirus transfection technology. The results of the CCK8 test showed that the proliferation rate of HeLa cells overexpressing Fra-1 was significantly reduced compared to that of vector group (Figure 1A). Furthermore, Fra-1–overexpressing HeLa cells formed fewer colonies than did the control cells (Figures 1B,C). Thus, these assays demonstrated that Fra-1 overexpression inhibited HeLa cell proliferation. We also examined the effect of Fra-1 overexpression on the cell cycle distribution of HeLa cells by flow cytometry and found that the percentage of cells in S phase was 13.32% among Fra-1–overexpressing HeLa cells, which was lower than that of 24.57% among the control cells (Figure 1D). Therefore, we conclude that Fra-1 overexpression significantly induced S-phase arrest in HeLa cells. Furthermore, we detected the effect of Fra-1 overexpression on cell apoptosis by flow cytometry with Hoechst 33342/PI double staining. The results showed that the proportion of early apoptotic cells among the Fra-1–overexpressing HeLa cells was 6.01%, while that in the control group was only 2.99% (Figure 1E). Thus, overexpression of Fra-1 promoted apoptosis among HeLa cells.

### Fra-1 Up-Regulated STAT1 and Altered p53 Signaling in Cervical Cancer Cells

**FIGURE 2:**
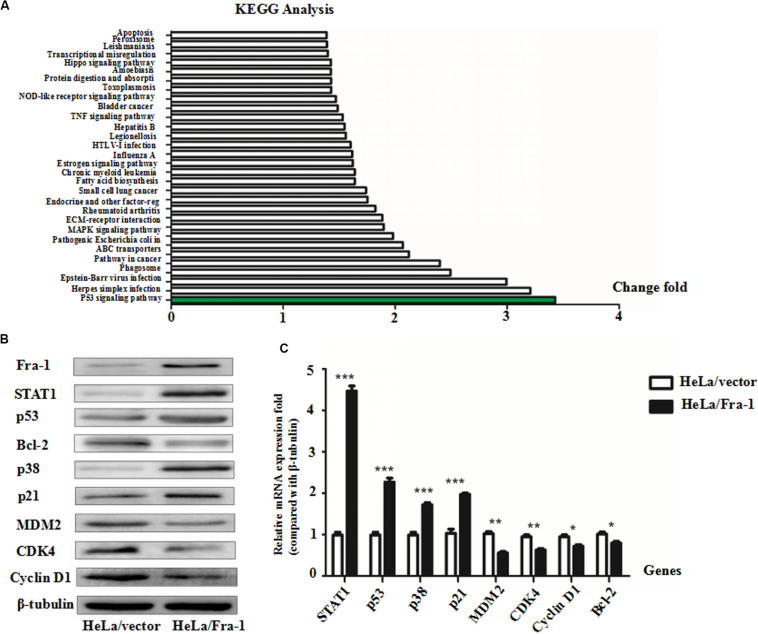
Fra-1 regulated p53 signaling pathway activity in cervical cancer cells. **(A)** mRNA chip analysis in HeLa/vector and HeLa/Fra-1 cells revealed differences in mRNA expression between the two groups. Differentially active signaling pathways were identified by gene ontology analysis. **(B)** Western blotting analysis of signal transducer and activator of transcription 1 (STAT1), p53, Bcl-2, p38, p21, MDM2, CDK4, and cyclinD1 protein levels in HeLa/vector and HeLa/Fra-1 cells. **(C)** Real-time polymerase chain reaction (PCR) analysis the mRNA expression levels of STAT1, p53, Bcl-2, p38, p21, MDM2, CDK4, and cyclinD1 in HeLa/vector and HeLa/Fra-1 cells. *P < 0.05; **P < 0.01; ***P < 0.001, *n* = 3.

To explore the mechanism by which Fra-1 overexpression altered key activities of cervical cancer cells, we performed the real-time PCR and western blot analyses to examine the effects on STAT1 expression and performed an mRNA microarray analysis to identify differentially activated signaling pathways. Our results showed that STAT1 protein was significantly up-regulated in Fra-1–overexpressing HeLa cells compared with the control cells, and the results of real-time PCR for STAT1 gene expression were consistent. Through Kyoto Encyclopedia of Genes and Genomes (KEGG) analysis, we observed obvious differences in signal pathway activity between Fra-1–overexpressing HeLa cells and control cells (Figure 2A). The p53 pathway was specifically identified and is strongly linked to the proliferation and apoptosis of cancer cells. Previous research has shown that STAT1 regulates the p53 activity and induces phosphorylation of p53 (McDermott et al., 2005). Therefore, we further tested the expression levels of key molecules involved in the p53 signaling pathway. We found that p53 was significantly up-regulated in Fra-1–overexpressing HeLa cells compared with the control cells, and p38 (MAPK) and p21 also were up-regulated in Fra-1–overexpressing HeLa cells. Conversely, MDM2, Bcl-2, CDK4, and cyclin D1 were down-regulated in Fra-1–over-expressing HeLa cells (Figure 2B). To confirm the differential expression of these proteins, we also detected the expression of the corresponding genes by real-time PCR. The results confirmed that p53, p38, and p21 were up-regulated at the mRNA level, while MDM2, Bcl-2, CDK4, and cyclin D1 were down-regulated at the mRNA level in Fra-1–over-expressing HeLa cells compared with the control cells (Figure 2C).

### Fra-1 Overexpression Restored Mitochondrial Disorder in Cervical Cancer Cells

**FIGURE 3:**
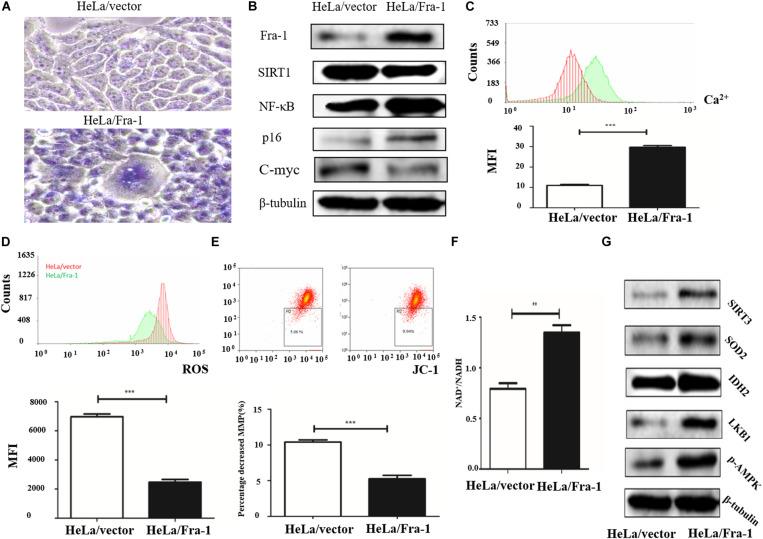
Fra-1 promoted cell senescence and restored mitochondrial disorder in cervical cancer cells. **(A)** β-Galactosidase assay for cell senescence in HeLa/vector and HeLa/Fra-1 cells. **(B)** Western blot analysis of Fra-1, SIRT1, NF-κB, p16, and c-Myc protein expression in HeLa/vector and HeLa/Fra-1 cells. **(C)** Flow cytometric analysis of intracellular Ca^2+^ concentration. Red color represents HeLa/vector group and green color represents HeLa/Fra-1 group. **(D)** Flow cytometric analysis of intracellular reactive oxygen species (ROS) concentration. Red color represents HeLa/vector group and green color represents HeLa/Fra-1 group. **(E)** Flow cytometric analysis of mitochondrial membrane potential (Δψm) in HeLa/vector and HeLa/Fra-1 cells. **(F)** NAD^+^/NADH ratio in HeLa/vector and HeLa/Fra-1 cells. **(G)** Western blot analysis of sirtuin 3 (SIRT3), SOD2, IDH2, LKB1, and p-AMPK expression in HeLa/vector and HeLa/Fra-1 cells. **P < 0.01; ***P < 0.001, *n* = 3.

To further study the effect of Fra-1 on cervical cancer cell senescence, we simulated the aging process of normal cells. Taking advantage of the reproductive senescence characteristic of the cells, we cultured the primary cells until they experienced senescence using the method of serial sub cultivation, and we finally used the 30th generation of cells for experiments evaluating mitochondrial disorder. Cell senescence was measured using a β-galactosidase staining assay, and the results showed that Fra-1 promoted HeLa cell senescence (Figure 3A). We also investigated whether Fra-1 affected the expression of senescence-related molecules and observed a significant reduction in SIRT1 expression in Fra-1–overexpressing HeLa cells. p65 is a key regulator of the process of senescence, and SIRT1 inhibits NF-κB activity mainly by deactivating p65. Our results showed that Fra-1 overexpression promoted the expression of NF-κB and p16, but decreased c-Myc expression in HeLa cells (Figure 3B).

Disturbance of intracellular calcium homeostasis can affect mitochondrial membrane potential and result in tumorigenesis (Vasileiou et al., 2019). Thus, we investigated whether the intracellular Ca^2+^ concentration is altered in Fra-1–overexpressing HeLa cells. We found a higher Ca^2+^ concentration in Fra-1–overexpressing HeLa cells than in control cells, as demonstrated by the mean fluorescence intensity (MFI) data (Figure 3C). ROS play crucial roles in many cell biological functions and in mitochondria. We examined the effect of Fra-1 overexpression on ROS levels and observed a significant decrease in the ROS level in Fra-1–overexpressing HeLa cells compared with that in control cells, with a corresponding decrease in the MFI from 6,973 ± 181.9 to 2,478 ± 175.1 (Figure 3D). A reduction in mitochondrial membrane potential (Δψm) is considered to be an early event during apoptosis and metabolism. Therefore, we also examined the mitochondrial membrane potential in Fra-1–overexpressing HeLa cells by JC-1 staining. A decrease in the mitochondrial membrane potential during apoptosis was represented by a decrease in red fluorescence intensity and an increase in green fluorescence intensity. Among the Fra-1–overexpressing HeLa cells, the percentage of cells with Δψm loss was greater than that among the control cells. The percentage decrease in the mitochondrial membrane potential was 9.94% with Fra-1 overexpression compared with 5.06% in control cells (Figure 3E).

We next investigated the possible mechanism for the effects of Fra-1 overexpression on mitochondrial function. It is well known that during oxidative phosphorylation, the electron transport chain in the mitochondrial inner membrane converts NADH to NAD^+^ through five complexes and produces oxygen and ATP. Thus, an increased NAD^+^/NADH ratio represents an increase in mitochondrial function. We found that the NAD^+^/NADH ratio was increased in Fra-1–overexpressing HeLa cells (Figure 3F). Western blot analysis revealed elevated expression of SIRT3, MnSOD2, and IDH2 in Fra-1–overexpressing HeLa cells (Figure 3G). Together these results revealed that Fra-1 overexpression restored mitochondrial function in HeLa cells.

### Fra-1 Suppressed the Warburg Effect in Cervical Cancer Cells

**FIGURE 4:**
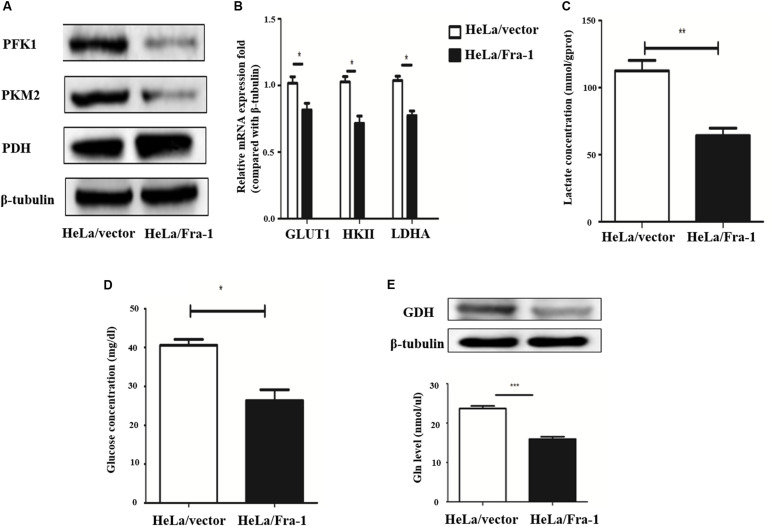
Fra-1 suppressed metabolic reprogramming in cervical cells. **(A)** Western blot analysis of the expression of key glycolysis enzymes PFK1, PKM2, and PDH in HeLa/vector and HeLa/Fra-1 cells. **(B)** Real-time polymerase chain reaction (PCR) analysis of the expression of glycolysis enzymes GLUT1, HK II, and LDHA in HeLa/vector and HeLa/Fra-1 cells. **(C)** Lactate concentration in HeLa/vector and HeLa/Fra-1 cells. **(D)** Glucose concentration in HeLa/vector and HeLa/Fra-1 cells. **(E)** Glutamine dehydrogenase (GDH) protein level and glutamine concentration in HeLa/Fra-1 and HeLa/vector cells. *P < 0.05; **P < 0.01; ***P < 0.001, *n* = 3.

The Warburg effect is a key characteristic of cancer cell metabolism, in which cancer cells are more inclined to glycolysis and glutamine metabolism. Therefore, we explored whether Fra-1 overexpression affected the Warburg effect in HeLa cells. First, we detected the effects of Fra-1 overexpression on the expression levels of key enzymes involved in aerobic glycolysis by Western blot and q-PCR analyses. The results showed that Fra-1 significantly inhibited the expression of rate-limiting enzymes in glycolysis, PFK1 and PKM2, while promoting the expression of PDH, which catalyzes pyruvate to produce acetyl-CoA (Figure 4A). Moreover, Fra-1 inhibited the expression of another glycolytic rate-limiting enzyme, HK II, in cervical cancer cells. Likewise, expression of glucose transporter 1 (Glut1), which is responsible for transporting glucose into cells, also was decreased in Fra-1-overexpressing HeLa cells. A significant reduction was observed in the mRNA level of LDHA, which converts pyruvate to lactate (Figure 4B). Finally, we examined the effect of Fra-1 overexpression on intracellular lactate levels and found less lactic acid accumulation in Fra-1–overexpressing HeLa cells than in control cells (Figure 4C). We also confirmed that the glucose level was decreased in Fra-1–overexpressing HeLa cells (Figure 4D).

The Warburg effect is often accompanied by abnormal glutamine metabolism and fatty acid synthesis. Our western blot results showed that glutamine dehydrogenase (GDH), which drives glutamine entry into the TCA cycle as α-KG, was significantly down-regulated in Fra-1–overexpressing HeLa cells. Moreover, Fra-1 overexpression was associated with a decreased glutamine concentration (Figure 4E).

All of the results described above indicate that Fra-1 likely suppresses the Warburg effect and fatty acid synthesis while also enhancing mitochondrial function and repairing mitochondrial dysfunction. Overall, Fra-1 plays key roles in the metabolic reprogramming of cervical cancer cells.

### Silencing of STAT1 Impaired the Inhibitory Effect of Fra-1 on Cervical Cancer Cell Proliferation

**FIGURE 5:**
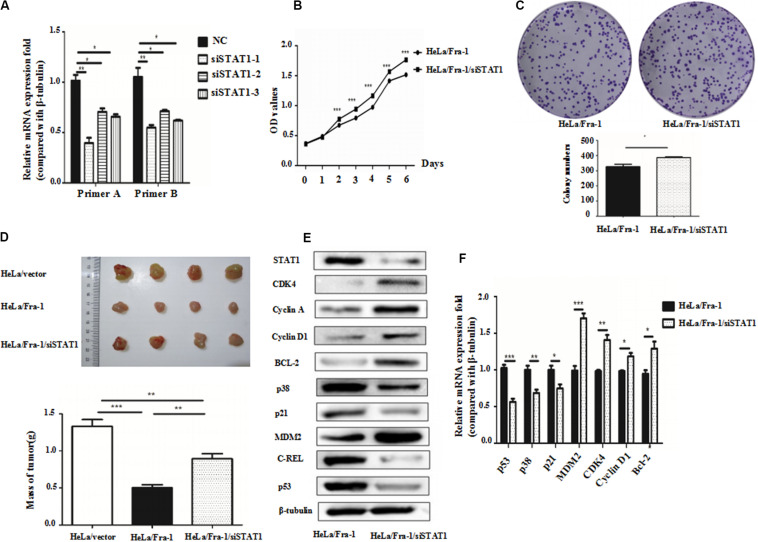
Silencing of signal transducer and activator of transcription 1(STAT1) by small interfering RNA promoted proliferation of cervical cancer cells via activation of the p53 signal pathway. **(A)** Real-time polymerase chain reaction (PCR) analysis of the silencing effects of three siRNAs for STAT1 in HeLa/Fra-1 cells. **(B)** Cell viability of HeLa/Fra-1 and HeLa/Fra-1/siSTAT1 cells according to CCK8 assay. **(C)** Colony forming ability of HeLa/Fra-1 and HeLa/Fra-1/siSTAT1 cells. **(D)** *In vivo* analysis of subcutaneous implanted tumor growth to compare the tumorigenic abilities of HeLa/vector, HeLa/Fra-1, and HeLa/Fra-1/siSTAT1 cells in mice. **(E)** Western blot analysis of protein expression of STAT1, CDK4, cyclin A, cyclin D1, p53, Bcl-2, c-REL, p38, p21, and MDM2 in HeLa/Fra-1 and HeLa/Fra-1/siSTAT1 cells. **(F)** Real-time PCR analysis of mRNA expression of STAT1, CDK4, cyclin D1, p53, Bcl-2, p38, p21, and MDM2 in HeLa/Fra-1 and HeLa/Fra-1/siSTAT1 cells. *P < 0.05; **P < 0.01; ***P < 0.001, *n* = 3.

Because we found that Fra-1 inhibited HeLa cell proliferation and up-regulated STAT1 expression, we investigated whether silencing STAT1 using siRNAs designed specifically for STAT1 could alter the inhibitory effect of Fra-1 on cervical cancer cell growth. Upon silencing of STAT1 in Fra-1–overexpressing HeLa cells, we found that the proliferation rate of Fra-1–overexpressing HeLa cells was restored based on CCK8 assay results as well as the colony forming assay (Figures 5A–C).

To verify the observed effects of STAT1 silencing *in vivo*, we compared the growth of tumors formed by HeLA/vector, HeLa/Fra-1, and HeLa/Fra-1/siSTAT1 cells in BALB/c-nu mice Tumor formation was significantly diminished in the HeLa/Fra-1 group compared with that in the HeLa/vector control group. However, tumorigenicity was significantly increased for HeLa/Fra-1/siSTAT1 cells. These findings suggest that Fra-1 inhibited the tumorigenicity of HeLa cells in nude mice via STAT1 (Figure 5D).

To explore the possible mechanism underlying the observed phenotypic changes, we analyzed the expression levels of several molecules closely related to the cell cycle distribution and apoptosis by western blotting and real-time PCR experiments. The results showed that, compared with the corresponding expression levels in Fra-1–overexpressing HeLa cells, the changes in p53, Bcl-2, p38, p21, MDM2, CDK4, and cyclin D1 expression were reversed by silencing of STAT1 in Fra-1–overexpressing HeLa cells. Specifically, p53, p38, and p21 were down-regulated at the protein level, and MDM2, CDK4, Bcl-2, and cyclin D1 were up-regulated at both the mRNA and protein levels after silencing of STAT1. The real-time PCR analysis revealed similar trends. Moreover, the expression levels of some of these proteins were even renewed to their levels in control HeLa cells (Figures 5E,F).

Together our results demonstrated that silencing of STAT1 impaired the inhibitory effect of Fra-1 on cervical cancer cell growth.

### Silencing of STAT1 Reversed the Effect of Fra-1 on Cell Senescence and Mitochondrial Dysfunction in Cervical Cancer Cells

**FIGURE 6:**
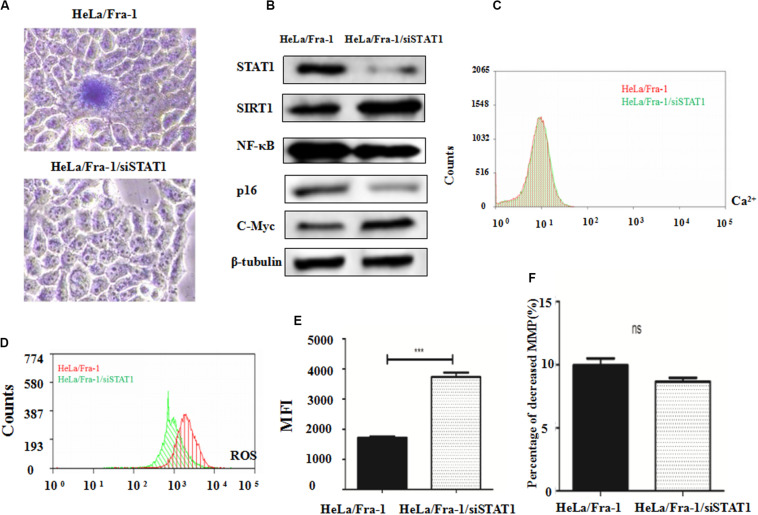
Silencing of signal transducer and activator of transcription 1 (STAT1) inhibited senescence and promoted mitochondrial dysfunction in cervical cancer cells. **(A)** β-Galactosidase assay for cell senescence in HeLa/Fra-1 and HeLa/Fra-1/siSTAT1 cells. **(B)** Western blot analysis of protein expression of c-Myc, SIRT1, NF-κB, and p16 in HeLa/Fra-1 and HeLa/Fra-1/siSTAT1 cells. **(C)** Flow cytometric analysis of intracellular Ca^2+^ concentration. Red color represents HeLa/Fra-1 group and green color represents HeLa/Fra-1/siSTAT1 group. **(D)** Flow cytometric analysis of intracellular reactive oxygen species (ROS) levels. Red color represents HeLa/Fra-1 group and green color represents HeLa/Fra-1/siSTAT1 group. **(E)** MFI data reflecting changes in the intracellular ROS concentration in HeLa/Fra-1 and HeLa/Fra-1/siSTAT1 cells. **(F)** Flow cytometric analysis of mitochondrial membrane potential (Δψm) in HeLa/Fra-1 and HeLa/Fra-1/siSTAT1 cells. *P < 0.05; **P < 0.01; ***P < 0.001, *n* = 3.

β-galactoside staining revealed that STAT1 silencing inhibited the senescence of Fra-1–overexpressing HeLa cells, restoring it to almost the background level (Figure 6A). These results indicate that STAT1 promoted senescence in Fra-1–overexpressing HeLa cells. We next explored STAT1 silencing affected the expression of senescence-related molecules in cervical cancer cells. Western blot analysis showed that the expression levels of SIRT1 and c-Myc oncogene were significantly up-regulated with STAT1 silencing, whereas the expression levels of p16 and NF-κB were down-regulated (Figure 6B). We detected changes in staining for Ca^2+^, ROS, and JC-1 in Fra-1–overexpressing HeLa cells with STAT1 silencing by flow cytometry. The results showed that STAT1 silencing did not change the level of the important intracellular messenger Ca^2+^ in Fra-1–overexpressing HeLa cells (Figure 6C). However, the ROS level was increased upon STAT1 silencing (Figure 6D), with the MFI data indicating an obvious increase in ROS (Figure 6E). JC-1 staining showed that STAT1 silencing had little effect on the mitochondrial membrane potential (Δψm) in Fra-1–overexpressing HeLa cells (Figure 6F).

### Silencing of STAT1 Recovered Metabolic Reprogramming in Cervical Cancer Cells

**FIGURE 7:**
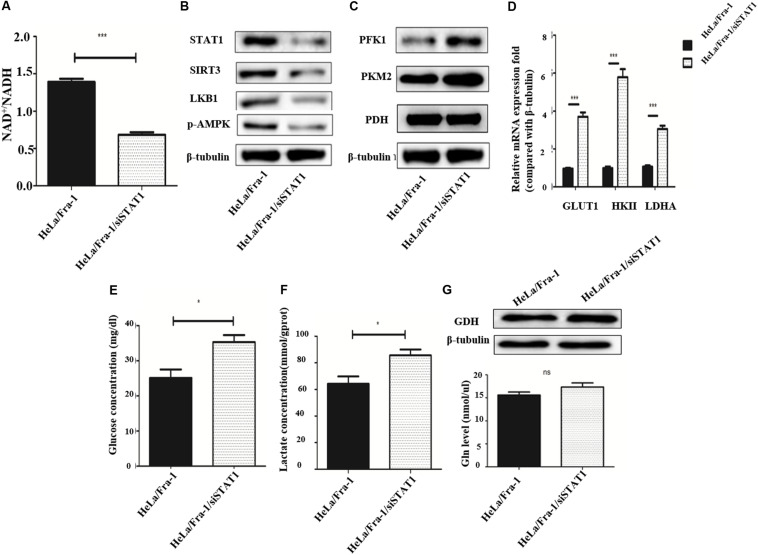
Silencing of signal transducer and activator of transcription 1(STAT1) recovered metabolic reprogramming in cervical cancer cells overexpressing Fra-1. **(A)** NAD + /NADH ratio in HeLa/Fra-1 and HeLa/Fra-1/siSTAT1 cells. **(B)** Western blot analysis of STAT1, sirtuin 3 (SIRT3), LKB1, and p-AMPK expression in HeLa/Fra-1 and HeLa/Fra-1/siSTAT1 cells. **(C)** Western blot analysis of the expression of key glycolysis enzymes PFK1, PKM2, and PDH in HeLa/Fra-1 and HeLa/Fra-1/siSTAT1 cells. **(D)** Real-time polymerase chain reaction (PCR) analysis of mRNA expression of glycolysis enzymes Glut1, HK II, and LDHA in HeLa/Fra-1 and HeLa/Fra-1/siSTAT1 cells. **(E)** Glucose concentration in HeLa/Fra-1 and HeLa/Fra-1/siSTAT1 cells. **(F)** Lactate concentration in HeLa/Fra-1 and HeLa/Fra-1/siSTAT1 cells. **(G)** Protein expression of glutamine dehydrogenase (GDH) and glutamine concentration in HeLa/Fra-1 and HeLa/Fra-1/siSTAT1 cells. NS: No statistical difference; *P* < 0.05; **P < 0.01; ***P < 0.001, *n* = 3.

We first examined the effect of STAT1 silencing on mitochondrial metabolism in Fra-1–overexpressing HeLa cells. The NAD^+^/NADH ratio was decreased obviously by STAT1 silencing (Figure 7A), indicating that STAT1 caused mitochondrial dysfunction in HeLa cells. After silencing of STAT1, the major mitochondrial deacetylase SIRT3 was down-regulated, and the expression of the crucial anti-oncogene LKB1 was significantly reduced, which thereby inhibited the activity of AMPK (Figure 7B). The reduction in AMPK expression further led to an increase in ACC, a rate-limiting enzyme for fatty acid synthesis.

Then we tested the influence of STAT1 silencing on the Warburg effect. The expression of PFK1 and PKM2, the rate-limiting enzymes of glycolysis, were shown to be decreased significantly (Figure 7C), and at the mRNA level, the change in HK II expression that occurred with Fra-1 overexpression was significantly reversed after silencing STAT1. In addition, the expression levels of glycolytic enzymes GLUT1 and LDHA were also significantly restored (Figure 7D). Further experiments showed that silencing of STAT1 significantly promoted the uptake of glucose in Fra-1–overexpressing HeLa cells (Figure 7E), and the intracellular content of lactate also was increased (Figure 7F). However, silencing of STAT1 had little effect on GDH in HeLa cells. The glutamine concentration also showed a slight increase in Fra-1–overexpressing cells (Figure 7G). These results showed that STAT1 silencing recovered mitochondrial dysfunction in HeLa cells and reversed the Warburg effect caused by Fra-1.

In summary, our results showed that Fra-1 inhibited cervical cancer cell growth and repaired metabolic dysfunction via STAT1, and these effects involved the Warburg effect and fatty acid metabolism in cervical cancer cells.

## Discussion

The Fra-1 and Jun proteins form heterodimers, resulting in a leucine zipper protein activator protein 1 (AP-1; Tolza et al., 2019). Fra-1 was shown to be closely related to the tumorigenesis of many tumors (He et al., 2015; Oliveira-Ferrer et al., 2015), and additional research identified Fra-1 as an oncogene-encoded transcription factor plays a crucial role in cancer invasion and metastasis (Luo et al., 2010). However, Fra-1 is down-regulated in the tumorigenic cell lines CGL3 and HeLa compared to non-tumorigenic cells, and it specifically inhibits the tumorigenicity of cervical carcinoma cell lines (Kehrmann et al., 2013). Our previous study showed that Fra-1 is expressed at low levels in cervical carcinoma tissues compared to adjacent normal tissues (Xiao et al., 2015). Therefore, the role of Fra-1 in cervical carcinoma needed to be investigated further.

The results of the present study show that Fra-1 overexpression inhibited cell proliferation, while promoting cell apoptosis and senescence in HeLa cervical cancer cells. Moreover, Fra-1 overexpression in these cells inhibited subcutaneous tumor formation in our *in vivo* experiment. Overall, Fra-1 overexpression in cervical cancer cells exerted a tumor suppressing effect based on our *in vitro* analyses of cell proliferation, cell cycle distribution, apoptosis, and senescence as well as in our xenograft tumor model.

Metabolic reprogramming is required to meet the different metabolic needs of cancer cells during tumorigenesis (Allison et al., 2017). Altered metabolism is a major feature of tumor cells, commonly including the Warburg effect and up-regulation of glutamine metabolism to convert carbon sources (Mills and O’Neill, 2016). In addition, mitochondrial function is often impaired, and mitochondrial metabolism is weakened. Mitochondria produce ATP via oxidative phosphorylation using pyruvate and fatty acids (Li et al., 2017). Cells with damaged mitochondria cannot generate enough ATP from mitochondrial oxidative phosphorylation and then are forced to rely on glycolysis for ATP generation (Lavie et al., 2018). In the present study, we explored whether Fra-1 caused these metabolic changes in HeLa cells. The mitochondrial membrane potential, Ca^2+^ concentration, and ROS level are closely related to cell metabolism. Changes in mitochondrial membrane potential modulate the activity of important enzymes in mitochondria, and the mitochondrial membrane potential, or ΔΨ, is a reflection of mitochondrial metabolic status (Arfaoui et al., 2018). Additionally, Ca^2+^ regulates cell metabolism together with ROS (Zhou et al., 2017). Our results indicated that Fra-1 overexpression increased the intracellular Ca^2+^ concentration, but decreased the ROS level and mitochondrial membrane potential in HeLa cells. These changes indicate that Fra-1 was related to metabolic changes in cervical cancer cells, and thus, we next sought to understand how Fra-1 causes metabolic reprogramming.

Sirtuin 3, a member of the NAD^+^-dependent deacetylases, is an important deacetylase in mitochondria. SIRT3 inhibits ROS production through two important target molecules, IDH2 and SOD2 (Yamato et al., 2016). Therefore, we examined the effect of Fra-1 overexpression on SIRT3, IDH2, and SOD2 expression in cervical cancer cells. We found that Fra-1 overexpression restored mitochondrial function in cervical cancer cells via the SIRT3 signaling pathway, and Fra-1 increased the expression of IDH2 and SOD2. The results indicated that Fra-1 up-regulated the expression of SIRT3, which led to changes in IDH2 and SOD2 expression as downstream molecules, and finally reduced the ROS content in cervical cancer cells. NAD^+^ and NADH are two coenzymes necessary for cellular energy metabolism. A low NAD^+^/NADH ratio leads to metabolic imbalance, and an increase in the NAD^+^/NADH ratio can ameliorate the metabolic imbalance (Pillai et al., 2010). Our results showed that Fra-1 increased the NAD^+^/NADH ratio in cervical cancer cells, indicating that Fra-1 repaired some functions of mitochondrial in HeLa cells.

Sirtuin 3 deacetylates and activates LKB1, thereby augmenting the activity of the LKB1-AMPK pathway (Li et al., 2015). Multiple studies have identified LKB1 as a tumor suppressor gene and shown that it inhibits important metabolic pathways in cancer cells by activating AMPK (Hawley et al., 2016; Blackmore et al., 2017). AMPK is an important energy receptor in cells that regulates and maintains energy homeostasis. Activated AMPK rapidly inactivates the fatty acid synthesis rate-limiting enzyme, ACC, thereby inhibiting lipid synthesis and reducing energy consumption (Lu et al., 2018). We found that Fra-1 promoted the expression of LKB1 and p-AMPK, which in turn reduced the expression of ACC.

We next wanted to explore the effect of Fra-1 overexpression on the Warburg effect. The Warburg effect indicates that during glycolysis, glycolysis is enhanced in tumor cells and the expression of metabolic enzymes and intermediates is increased. We found Fra-1 overexpression down-regulated glycolytic rate-limiting enzymes PFK1, PKM2, and HK II as well as the expression of important glycolytic enzymes GLUT1, LDHA, and pyruvate dehydrogenase kinase. Additionally, Fra-1 overexpression led to decreased glucose and lactate concentrations in cervical cancer cells. The Warburg effect also increases glutamine and lipid metabolism, and our results showed that Fra-1 overexpression decreased the glutamine concentration as well as the expression of GDH in cervical cancer cells. In conclusion, in the present study, Fra-1 repaired mitochondrial metabolism in cervical cancer cells, but inhibited the Warburg effect and lipid metabolism.

Signal transducer and activator of transcription 1 is an important transcription factor that has repeatedly been shown to be a tumor suppressor. STAT1 inhibits cell proliferation through immune regulation and other mechanisms (Zhang et al., 2017). Changes in the expression level of STAT1 can alter the proliferation of HeLa cell (Lei et al., 2015). We found that Fra-1 overexpression in cervical cancer cells caused very significant changes in STAT1 expression at both the protein and mRNA levels. Therefore, we explored whether the transcription factor STAT1 is involved in the effects of Fra-1 in cervical cancer cells. We found that after silencing of STAT1, the tumor suppression effect of Fra-1 overexpression disappeared through a series of *in vivo* and *in vitro* experiments. Silencing of STAT1 restored the proliferation of cervical cancer cells and the growth of xenograft tumors to some extent. The expression levels of some important molecules in the p53 signaling pathway also were altered by STAT1 silencing in Fra-1–overexpressing HeLa cells. We also investigated the effects of STAT1 on metabolic reprogramming of cervical cancer cells overexpressing Fra-1. Our results revealed that STAT1 silencing recovered the ROS level in Fra-1–overexpressing cervical cancer cells and reduced the NAD^+^/NADH ratio, which increased the metabolic instability of the cervical cancer cells. Additionally, STAT1 silencing up-regulated the expression of glycolysis enzymes PFK1, PKM2, HK II, GLUT1, and LDHA as well as increased the concentrations of glycolysis products glucose and lactate. After silencing of STAT1, the expression levels of SIRT3, LKB1 and p-AMPK also were reduced. The decrease of p-AMPK expression can increase the expression of the rate-limiting enzyme ACC in fatty acid synthesis, and this was observed in our experiment. In conclusion, silencing of STAT1 caused metabolic imbalance and recovered the Warburg effect in Fra-1–overexpressing cervical cancer cells.

In summary, our results showed that Fra-1 overexpression in cervical cancer cells inhibited cell growth and repaired metabolic dysfunction, including the Warburg effect and fatty acid metabolism, via STAT1 regulation of p53 signaling.

## Data Availability Statement

The original contributions presented in the study are included in the article/supplementary material, further inquiries can be directed to the corresponding authors.

## Ethics Statement

The animal study was reviewed and approved by the Committee of the Use of Live Animals in Teaching and Research at the Central South University.

## Author Contributions

YZ and SX conceived and supervised the study. MZ completed the main experiments of cell proliferation, ROS, lactic acid detection and cervical cancer tumor growth *in vivo*. LL performed the experiments of NAD^+^/NADH, cell cycle, and cell apoptosis. JH and MG performed the experiments of western blot. ZH performed the experiments of JC-1 and Intracellular Ca^2+^ concentration assay. CY and XJ performed the experiments of siRNAs and measurement of glucose concentration and analyzed data. MZ wrote the manuscript. YZ made manuscript revisions. All authors reviewed the manuscript.

## Conflict of Interest

The authors declare that the research was conducted in the absence of any commercial or financial relationships that could be construed as a potential conflict of interest.

## Abbreviations

CDK4: cyclin-dependent kinase 4
FOSL1 (also known as Fra-1): FOS-like antigen 1
GDH: glutamine dehydrogenase
IDH2: isocitrate dehydrogenase 2
MDM2, MDM2 proto-oncogene: E3 ubiquitin protein ligase
MnSOD2: Mn superoxide dismutase 2
PKF: G6-phosphofructokinase
PKM2: pyruvate kinase muscle isozyme 2
SIRT3: sirtuin 3
STAT1: signal transducer and activator of transcription 1
TP53: tumor protein p53.
